# Trends in gynecological cancer incidence, mortality, and survival among elderly women: A SEER study

**DOI:** 10.1002/agm2.12297

**Published:** 2024-04-29

**Authors:** Subhadra Priyadarshini, Prafulla Kumar Swain, Khushi Agarwal, Diptismita Jena, Sourav Padhee

**Affiliations:** ^1^ Department of Statistics Utkal University Bhubaneswar Odisha India; ^2^ Research and Development Department Kalinga Institute of Medical Sciences (KIMS) Bhubaneswar Odisha India; ^3^ Department of Statistics Ravenshaw University Cuttack Odisha India

**Keywords:** AAPC, annual percentage change (APC), elderly women, gynecological cancer, SEER

## Abstract

**Objectives:**

This paper aims to comprehensively analyze trends in gynecological cancers among elderly women in the United States from 1975 to 2020.

**Methods:**

Surveillance, Epidemiology, and End Results (SEER) population data were utilized for the analysis. Annual Percentage Change (APC) and Average APC were estimated using join‐point regression to assess trends in mortality rates.

**Results:**

The study reveals an increasing pattern of incidence and mortality in all gynaecological cancer sites except cervical cancer among elderly. The incidence of cervical cancer decreased from 1975 to 2007 and then increased, whereas cancer‐specific mortality decreased from 1977 to 2020, indicating positive advancements in detection and treatment.

**Conclusions:**

Despite progress in managing certain gynecological cancers, challenges persist, particularly evidenced by increasing mortality rates for cancers in other female genital organs. This underscores the necessity for sustained research efforts and targeted interventions to address these ongoing challenges effectively.

## Graphical abstract

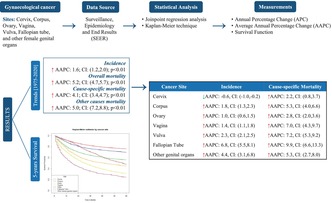

## 1 INTRODUCTION

Gynecological cancers encompass cancers affecting the cervix uteri, corpus uteri, ovaries, fallopian tubes, vulva, and vagina. This category presents a significant public health challenge due to the persistent role of gynecological malignancies as a leading cause of cancer‐related mortality. The global burden of cancer incidence and mortality is escalating rapidly, driven by both population growth and aging. According to GLOBOCAN report, gynecological cancers contribute to nearly 40% of all cancer cases and over 30% of all cancer‐related deaths among women worldwide.^1^

Cervical cancer presently stands as the second most prevalent gynecological cancer, with 0.6 million new cases reported in 2020. It ranks fourth among causes of cancer‐related deaths in women, claiming approximately 342,000 lives globally.^2^ Ovarian cancer ranks fourth among gynecological malignancies, with approximately 313,959 new cases and 207,252 deaths in 2020. Uterine cancer is the third most diagnosed cancer in women worldwide, with an estimated 66,200 new cases and 13,030 deaths in the United States in 2023 (Siegel et al., 2023).^3^ Despite advancements in cancer research, the incidence of gynecological malignancies in the United States continues to rise. The factors contributing to this increase are likely multifactorial, heterogeneous, and poorly understood.

Several industrialized countries, including the United States, have witnessed an uptick in the incidence of gynecological cancers among women. A study by Knudsen et al.^4^ explored trends in incidence, mortality, and survival in gynecological cancers among elderly Danish women from 1980 to 2012. They concluded that mortality rate and survival are age‐dependant with significant shorter survival for the elderly. Piechocki et al.^5^ reported a significant increase in ovarian and corpus uteri cancer, as well as breast cancer, and a decrease in cervical and vaginal cancer incidence in Poland. A recent study in the United States by Somasegar et al.^6^ analyzed uterine cancer mortality trends over a 50‐year period, emphasizing age and race. They observed a sixfold higher uterine cancer mortality rate for patients aged 70 years or older compared to those aged 50 to 59 years.

Advancing age is a major risk factor for cancer, with individuals over 65 years accounting for 60% of newly diagnosed malignancies and 70% of all cancer deaths (Berger et al., 2006).^7^, ^8^ Elderly women are frequently diagnosed at later stages and experience worse outcomes than younger patients.^9^, ^10^ Consequently, there may be an unmet need among older women in terms of gynecological cancer prevention and treatment.^11^

The objective of this study is to utilize United States Surveillance, Epidemiology, and End Results (SEER) population data to analyze trends in the incidence and mortality of gynecological cancers among elderly women between 1975 and 2020.

A detailed and comprehensive analysis of gynecological cancer trends will aid policymakers in assessing the cancer burden, establishing health service infrastructures, and allocating public health resources.

## 2 METHODS

### 2.1 Data sources

Retrospective data for this study were sourced from the National Cancer Institute's SEER 8 registry database (November 2022 supplement, 1975–2020). Cancer data pertaining to the female genital system, encompassing all gynecological cancer sites (cervix, corpus, ovary, vagina, vulva, fallopian tube and other female genital organs), were extracted. The analysis has focused on women aged 65 years or above, excluding cases with nonclinical reporting sources (autopsy only, death certificate, nursing/convalescent home/hospital), missing cause of death information, or zero survival months.

The variables included in the analysis comprised age at first diagnosis, year of diagnosis, cancer site, survival months, vital status, and cause of death. Gynecological cancer‐specific mortality was the primary outcome, with other cause‐related deaths considered as the secondary endpoint. Patient status was determined by combining SEER cause‐specific death classification and SEER other cause of death classification variables. Year of mortality was obtained by adding the survival months with year of diagnosis.

### 2.2 Statistical analysis

Descriptive statistics have been presented for both continuous and categorical variables. The Kaplan–Meier technique was employed to estimate the survival function. Joinpoint regression analysis, utilizing the joinpoint regression program version 4.8.0.1 (Statistical Methodology and Applications Branch, Surveillance Research Program, National Cancer Institute, Bethesda, MD), was performed to identify trends.^12^ The number of joinpoints ranged between 0 and 3, allowing the selection of the best‐fitting model with the estimated annual percentage change (APC) for each segment. The average APC (AAPC) illustrated the trend over the entire period. When no change points were found, only one APC value represented the trendline for the whole period. The detail mathematical treatment of joinpoint regression is given in the following section.

### 2.3 Joinpoint regression model

Joinpoint regression analysis is being performed to identify the period in which any significant changes on cancer‐specific mortality rates for cervix, corpus, ovary, vagina, vulva, fallopian tube, and other female genital organs during 1975 to 2020.

Mathematically, the joinpoint regression model is used for modeling the observations {($x_{1},y_{1}),\ldots \ldots \ldots \ldots ,\left(x_{n},y_{n}\right)\}$, where $x_{1}\leq x_{2}\leq \cdots \cdots \cdots \leq x_{n}$. The simple linear join‐point regression model^13^ with *k* + 1 segments for *i* = 1, 2,…., *n*, can be written as:

(1) $$E\left(y|x\right)=\beta_{0}+\beta_{1}x+\delta_{1}{\left(x-\tau_{j}\right)}^{+}+\ldots +\delta_{k}{\left(x-\tau_{k}\right)}^{+}+\varepsilon_{i}=\beta_{0}+\beta_{1}x+\sum_{j=1}^{k}\delta_{j}\left(x-\tau_{j}\right)$$

Where ${\left(x-\tau_{k}\right)}^{+}=\left\{\begin{array}{c} \left(x-\tau_{k}\right),x>\tau_{k}\;\\ \;0,\text{Other wise}\; \end{array}\right.,\delta_{k}=\beta_{\left(k+1\right)}-\beta_{k}\;\text{and}\;\varepsilon_{i}\approx N\left(0,\sigma^{2}\right)$.

The APC and AAPC were proposed to summarize and compare the rate of changes of cancer‐specific mortality rates that occur over the given period of time and given by:

(2) $$\mathrm{APC}=\left(e^{\beta_{1}+\delta_{1}+\delta_{2}+\ldots +\delta_{j}}-1\right)\times 100$$

(3) $$\text{AAPC}=\left\{\exp\left(\frac{\sum w_{i}\beta_{i}}{\sum w_{i}}\right)-1\right\}\times 100$$

All statistical analyses were conducted using MS Excel and SPSS. A *P* value <0.05 was deemed statistically significant.

### 2.4 Ethical approval

As this data is available on public sources, i.e., the SEER database of the National Cancer Institute (http://seer.cancer.gov), no ethical clearance is required for this study.

## 3 RESULTS

The Table 1 shows the descriptive characteristics of the study population. The present study incorporated a cohort of 112,192 women, characterized by a median age of 75 years and median survival duration of 43 months after diagnosis. The racial composition of the study population was as follows: 4.6% Hispanic, 83.2% Non‐Hispanic White, 5.7% Non‐Hispanic Black, 5.8% Non‐Hispanic Asian or Pacific Islander, 0.5% Non‐Hispanic American Indian/Alaska Native, and 0.2% categorized as Non‐Hispanic Unknown Race.

**TABLE 1:** Descriptive characteristics of elderly gynecological cancer patients.

Variable	Category	Frequency	Percent
Age group	65–69	34,126	30.4
	70–74	28,476	25.4
	75–79	22,052	19.7
	80–84	15,084	13.4
	85–89	8493	7.6
	90+	3961	3.5
Year of diagnosis	1975–1980	10,381	9.3
	1981–1990	21,368	19.0
	1991–2000	24,219	21.6
	2001–2010	24,246	21.6
	2011–2020	31,978	28.5
Cancer site (ICD‐O‐32023 Revision)	Cervix	8545	7.6
	Corpus	63,215	56.3
	Ovary	28,191	25.1
	Vagina	2035	1.8
	Vulva	7339	6.5
	Fallopian tube	1971	1.8
	Other female genital organs	896	0.8
Survival time (in months) (Median)		43	
Vital status	Alive	26,377	23.5
	Death	85,815	76.5
Cause of death	Cancer‐specific	44,057	39.3
	Other cause	41,758	37.2

Over the course of 1975 to 2020, a noteworthy fourfold surge in the diagnosis of gynecological cancer cases among elderly women was observed. Uterine corpus cancers accounted for the majority, comprising 56.3% of the cases, followed by 25.1% in the ovary. Despite this, only 23.5% of the cases resulted in survival, while a substantial 76.5% faced mortality. Of this mortality, 39.3% was attributed to cancer.

**FIGURE 1:**
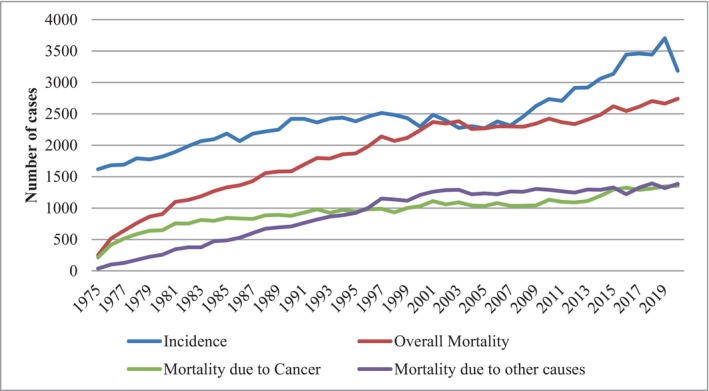
Trends in gynecological cancer incidence and mortality in elderly (1975–2020).

Figure 1 depicts the temporal trend in gynecological cancer incidence and mortality among elderly women spanning the years 1975 to 2020. Strikingly, all key indicators, including incidence, overall mortality, mortality attributed to cancer specific, and mortality due to other causes, have experienced a substantial and notable increase over this time frame.

The Table 2 shows the decadal trends of different gynecological cancers by age group. Over the span of 1975 to 2020, distinct patterns in the incidence of gynecological cancers have surfaced across varying age groups. Cervical cancer has exhibited an increase in incidence within the 65 to 69 and 70 to 74 age brackets. Uterine corpus cancer, on the other hand, has seen a noteworthy rise, particularly among women aged 85 and above, diverging from the trends in other age groups. Fallopian tube cancer incidence displayed an upward trajectory for all age groups, excluding the 70 to 74 and 85 to 89 cohorts. In the case of ovarian cancer, a decline in incidence is observed for age groups under 79, while a substantial increase is evident among women aged 80 and above. Similar trends are noted for vaginal and vulva cancers, with decreasing incidence in age groups under 80 and a significant rise in those aged 80 and above. Other genital female cancers exhibit a roughly analogous trend, albeit not identical across all age groups.

**TABLE 2:** Incidence of different gynecological cancers in past five decades by elderly age groups (in percent).

Site of cancer	Age at diagnosis	Year of diagnosis				
		1975–1980	1981–1990	1991–2000	2001–2010	2011–2020
Cervix	65–69	31.9	32.2	29.5	30.5	35.5
	70–74	25.2	24.7	23.4	23.8	26.4
	75–79	18.4	19.4	19.3	19.3	16.5
	80–84	14.4	11.2	15.2	14.2	10.2
	85–89	7.2	8.9	8.1	7.7	7.4
	90+	2.9	3.6	4.4	4.5	4
Corpus	65–69	38	34.6	26.7	30.6	38
	70–74	26.9	28	26.8	23.3	27.3
	75–79	17.7	19.6	22.4	19.7	16.7
	80–84	10.8	10.5	14.4	15.2	9.7
	85–89	5.1	5	7.1	7.9	5.6
	90+	1.5	2.3	2.5	3.4	2.7
Ovary	65–69	32	31	25.5	23.9	29.1
	70–74	26.2	27.5	25.7	23	24.1
	75–79	21.1	19.7	22.6	21.4	19.3
	80–84	13.1	13	15.3	17.2	14.4
	85–89	6.1	6.3	7.9	9.9	8.8
	90+	1.5	2.5	3.1	4.6	4.3
Vagina	65–69	25.4	20.1	17.9	17	24.8
	70–74	20.4	20.4	18.4	17.8	20.5
	75–79	21.9	22.5	26.3	20.4	19.5
	80–84	17.9	19.3	17.4	20.2	14.7
	85–89	10	8.8	13.8	15.5	12.4
	90+	4.5	8.8	6.1	9	8.1
Vulva	65–69	21.2	17.5	13.8	14	19.7
	70–74	22.2	19.3	20.2	16.7	19.4
	75–79	20.3	22.7	20.7	19.4	18.1
	80–84	19	17.6	19.9	21.4	17.1
	85–89	11.8	14.2	15.5	16.4	14.9
	90+	5.6	8.7	10.1	12.1	10.8
Fallopian tube	65–69	31.7	29.3	27.7	35.5	33.9
	70–74	33.3	28.7	33.5	24.2	31.4
	75–79	19	23.4	21.4	19.8	19.6
	80–84	9.5	9.6	13.4	14.2	10.3
	85–89	6.3	7.8	3.6	5.7	4.2
	90+	0	1.2	0.4	0.6	0.7
Other female genital organs	65–69	25	24.5	14.8	22.2	20
	70–74	23.6	24.5	21.3	17.5	20.5
	75–79	26.4	22.6	19.4	19	17.4
	80–84	8.3	13.2	20.4	17.5	17.6
	85–89	12.5	12.3	15.7	15.1	14.3
	90+	4.2	2.8	8.3	8.7	10.3

*Note*: Lowest to highest: 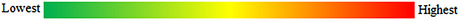

**FIGURE 2:**
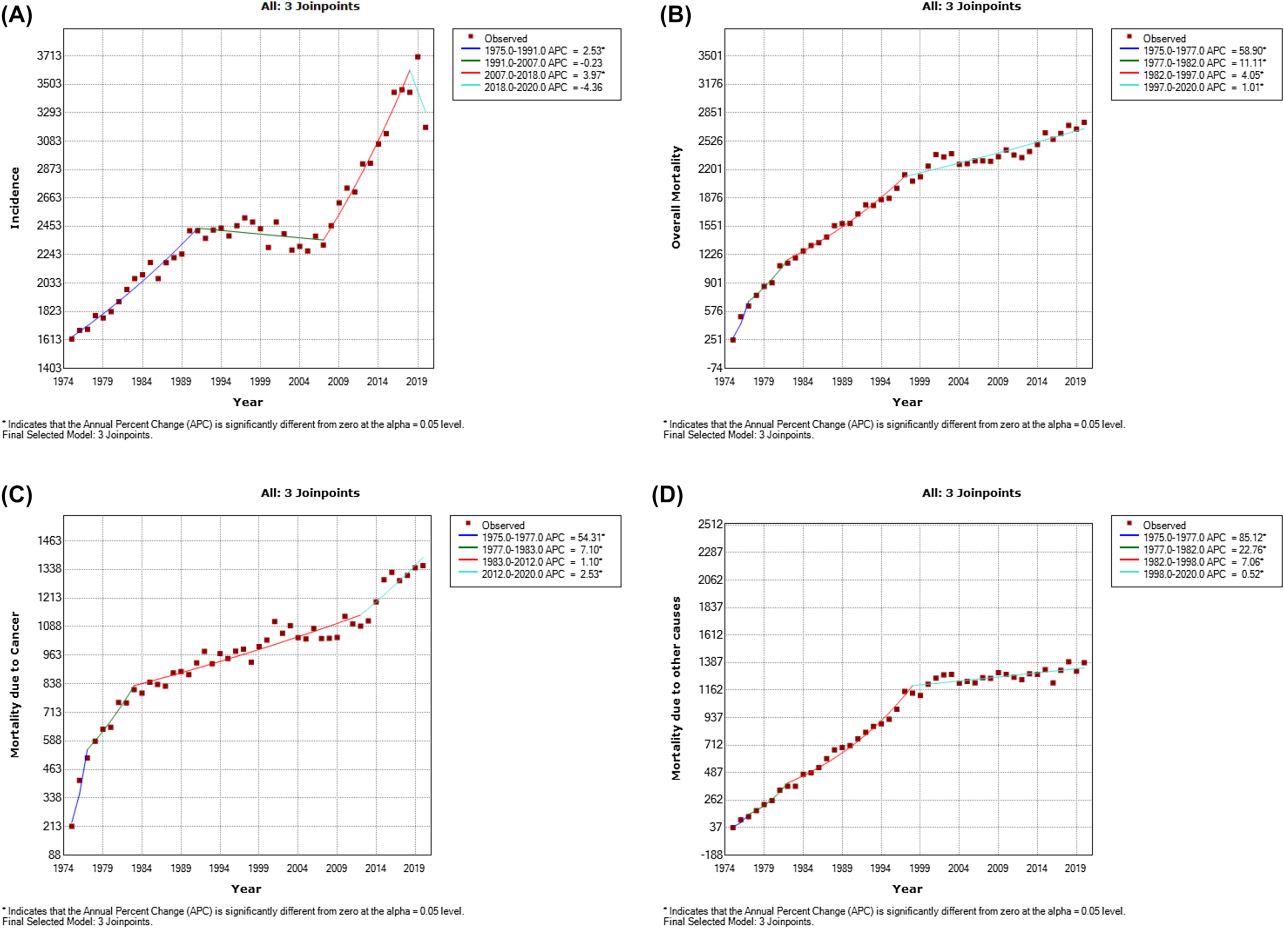
Joinpoint regression analysis of trends in incidence (A), overall mortality (B), mortality due to cancer (C), and mortality due to other causes (D).

Table 3 presents the APC obtained from joinpoint regression model. Here, the positive value of APC suggests an increasing trend and the negative value of APC suggests a decreasing trend. The analysis of gynecological cancer trends over different periods reveals significant insights into both incidence and mortality rates (Figure 2). Notably, the annual percentage change (APC) in incidence exhibited statistical significance during the periods 1975 to 1991 and 2007 to 2018, showcasing a 1.5 times increase. The AAPC in incidence also proved significant at 1.6%. Meanwhile, the overall mortality rate experienced a statistically significant APC throughout the study, showing a noteworthy decline from 58.9% in 1975 to 1977 to 1% in 1997 to 2020. The AAPC in overall mortality rate echoed significance at 5.2%. This positive trend extended to mortality rates due to cancer, revealing a 21.72% decrease in APC from 1975 to 1977 to 2012 to 2020, with a significant AAPC of 4.1%. Encouragingly, mortality rates due to other causes displayed a substantial reduction from 85.1% to 0.5%, accompanied by a noteworthy AAPC of 8%.

**TABLE 3:** Annual percentage changes in overall gynecological cancer incidence, mortality and cancer‐specific mortality among elderly women (1975–2020).

	Joinpoint	APC	95% CI of APC	Test statistic (*t*)	*P* value	AAPC	95% CI of AAPC
Incidence	1975–1991	2.5*	(2.2, 2.8)	17.5	<0.001	1.6*	(1.2, 2.0)
	1991–2007	−0.2	(−0.5, 0.1)	−1.4	0.157		
	2007–2018	4.0*	(3.4, 4.6)	13.4	<0.001		
	2018–2020	−4.4	(−11.3, 3.2)	−1.2	0.240		
Overall mortality	1975–1977	58.9*	(45.3, 73.8)	10.5	<0.001	5.2*	(4.7, 5.7)
	1977–1982	11.1*	(8.0, 14.3)	7.5	<0.001		
	1982–1997	4.0*	(3.6, 4.5)	19.2	<0.001		
	1997–2020	1.0*	(0.8, 1.2)	10.2	<0.001		
Mortality due to cancer	1975–1977	54.3*	(38.1, 72.4)	7.9	<0.001	4.1*	(3.4, 4.7)
	1977–1983	7.1*	(4.5, 9.8)	5.6	<0.001		
	1983–2012	1.1*	(0.9, 1.3)	12.1	<0.001		
	2012–2020	2.5*	(1.3, 3.8)	4.2	<0.001		
Mortality due to other causes	1975–1977	85.1*	(62.3, 111.2)	9.5	<0.001	8.0*	(7.2, 8.8)
	1977–1982	22.8*	(17.8, 28.0)	10	<0.001		
	1982–1998	7.1*	(6.5, 7.7)	24.9	<0.001		
	1998–2020	0.5*	(0.2, 0.8)	3.4	0.002		

* Indicates that the APC and AAPC are significantly different from zero at the *α* = 0.05 level).

**FIGURE 3:**
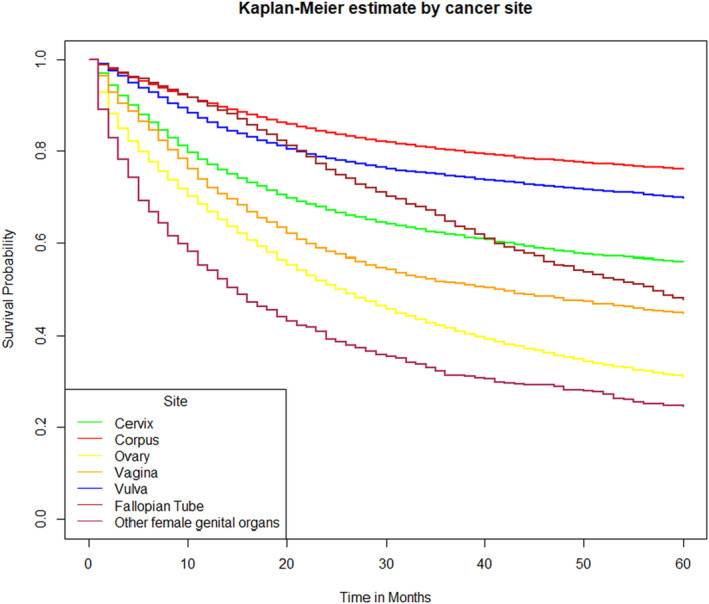
Kaplan–Meier Plot for 5‐year survival by cancer site.

From the above (Table 4) we can infer that gynecological cancers vary significantly in their survival rates. Uterine corpus cancer having a 76% survival rate, vulva cancer at 70%, and cervical cancer at 56% indicates differences in prognosis. All other types of gynecological cancers under consideration were found to have much lower 5‐year survival rates (Figure 3).

**TABLE 4:** 5 year survival of different gynecological cancers.

Site	5‐year survival	Std. Error	95% CI	
			Lower	Upper
Cervix	0.560	0.006	0.549	0.572
Corpus	0.761	0.002	0.757	0.764
Ovary	0.310	0.003	0.304	0.315
Vagina	0.448	0.001	0.125	0.474
Vulva	0.697	0.006	0.686	0.709
Fallopian tube	0.478	0.014	0.452	0.506
Other female genital organs	0.246	0.018	0.213	0.283

The data presented in Table 5 offers a comprehensive overview of the APC and AAPC in the incidence of different gynecological cancers spanning the years 1975 to 2020. Firstly, the incidence of cervical cancer displayed a notable shift, with the APC increasing significantly from −0.8% in 1975 to 1994 to 1.6% in 2007 to 2020, indicating a threefold rise. The AAPC for cervical cancer was also found to be statistically significant at −0.6%, underscoring a significant trend over the entire period. Similarly, uterine corpus cancer exhibited a noteworthy change in incidence, with the APC rising from 2.3% in 1975 to 1994 to 4.8% in 2006 to 2018. The AAPC for uterine corpus cancer was also found to be significant at 1%, emphasizing a sustained increase over the years. Ovary cancer demonstrated statistically significant APC in the period 1975 to 1990, and the AAPC for ovary cancer was significant at 1%, signifying a consistent trend in incidence. Furthermore, the incidence of vaginal, vulva, and fallopian tube cancers displayed both statistically significant APC and AAPC over the entire period under consideration (1975–2020), highlighting the importance of ongoing scrutiny and targeted interventions.

**TABLE 5:** APC and AAPC of incidence of different gynecological cancer, 1975 to 2020.

Site	Joinpoint	APC	95% CI of APC	Test statistic (*t*)	*P* value	AAPC	95% CI of AAPC
Cervix	1975–1994	−0.8*	(−1.3, −0.3)	−3.3	0.002	−0.6*	(−1.0, −0.2)
	1994–2007	−2.4*	(−3.4, −1.4)	−4.9	<0.001		
	2007–2020	1.6*	(0.7, 2.5)	3.6	0.001		
Corpus	1975–1994	2.3*	(2.0, 2.6)	15.8	<0.001	1.8*	(1.3, 2.3)
	1994–2006	−0.9*	(−1.6, −0.3)	−2.8	0.008		
	2006–2018	4.8*	(4.1, 5.5)	14.3	<0.001		
	2018–2020	−4.6	(−13.6, 5.2)	−1.0	0.332		
Ovary	1975–1990	3.8*	(3.2, 4.4)	13.3	<0.001	1.0*	(0.6, 1.5)
	1990–2016	0.2	(−0.1, 0.4)	1.3	0.201		
	2016–2020	−3.4	(−7.4, 0.8)	−1.7	0.107		
Vagina	1975–2020	1.4*	(1.1, 1.8)	8.7	<0.001	1.4*	(1.1, 1.8)
Vulva	1975–2020	2.3*	(2.1, 2.5)	20.6	<0.001	2.3*	(2.1, 2.5)
Fallopian Tube	1975–2006	3.2*	(2.2, 4.2)	6.4	<0.001	6.8*	(5.5, 8.1)
	2006–2020	15.3*	(11.6, 19.1)	8.8	<0.001		
Other Female Genital organs	1975–2004	−0.7	(−1.8, 0.5)	−1.1	0.271	4.4*	(3.1, 6.8)
	2004–2020	14.4*	(11.1, 17.8)	9.2	<0.001		

* Indicates that the APC and AAPC are significantly different from zero at the α = 0.05 level.

The above (Table 6) elucidates the APC and AAPC in cancer‐specific mortality rates for various gynecological cancers spanning the years 1975 to 2020. Beginning with cervical cancer, a remarkable decline in APC is observed, decreasing significantly from 88.9% in 1975 to 1977 to −0.6% in 1977 to 2020. The AAPC for cervical cancer mortality rate was also found to be significant at 2.2%, signifying a noteworthy sustained reduction over the entire duration. Similarly, uterine corpus cancer exhibited a substantial shift, with the APC in mortality rate dropping significantly from 72.7% in 1975 to 1977 to 4.2% in 2008 to 2020. The AAPC for uterine corpus cancer mortality rate was also found to be significant at 5.3%, indicating a notable decrease over the years. For ovarian cancer, the APC in mortality rate demonstrated a significant reduction from 36% in 1975 to 1977 to −0.5% in 2001 to 2020. The AAPC for ovarian cancer mortality rate was found to be significant at 2.8%, suggesting a sustained decrease over the study period. Moreover, the mortality rates for vaginal, vulvar, and fallopian tube cancers showed significant decreases in both APC and AAPC over the entire period under consideration, reflecting positive trends in the management of these cancers. Conversely, cancer in other female genital organs displayed a statistically significant APC of 19% during 2006 to 2020, underscoring the need for further investigation and intervention in this specific category.

**TABLE 6:** APC and AAPC of cancer‐specific mortality of different gynecological cancer, 1975 to 2020.

Site	Joinpoint	APC	95% CI of APC	Test statistic (*t*)	*P* value	AAPC	95% CI of AAPC
Cervix	1975–1977	88.9*	(36.7, 161.1)	4.0	<0.001	2.2*	(0.8,3.7)
	1977–2020	−0.6*	(−0.9, −0.4)	−4.6	<0.001		
Corpus	1975–1977	72.7*	(50.6, 98.0)	8.1	<0.001	5.3*	(4.0, 6.6)
	1977–1981	11.8*	(4.4, 19.7)	3.3	0.002		
	1981–2005	1.6*	(1.3, 1.9)	10.8	<0.001		
	2005–2008	−2.7	(−15.2, 11.5)	−0.4	0.683		
	2008–2020	4.2*	(3.4, 5.0)	10.3	<0.001		
Ovary	1975–1977	36.0*	(17.1, 58.0)	4.2	<0.001	2.8*	(2.0, 3.6)
	1977–1985	5.9*	(3.8, 8.1)	5.8	<0.001		
	1985–2001	1.7*	(1.1, 2.3)	5.4	<0.001		
	2001–2020	−0.5*	(−0.9, 0)	−2.1	0.042		
Vagina	1975–1977	197.5*	(66.5, 431.6)	3.8	<0.001	7.0*	(4.3, 9.7)
	1977–2020	2.0*	(1.5, 2.5)	7.9	<0.001		
Vulva	1975–1977	159.0*	(71.6, 290.9)	4.7	<0.001	7.2*	(5.3, 9.2)
	1977–2020	2.9*	(2.5, 3.3)	16.2	<0.001		
Fallopian Tube	1975–1979	51.5*	(12.8, 103.4)	2.9	0.007	9.9*	(6.6, 13.3)
	1979–2008	2.6*	(1.0, 4.1)	3.3	0.002		
	2008–2020	16.8*	(10.6, 23.4)	5.7	<0.001		
Other Female Genital organs	1975–2006	−0.3	(−2.4, 1.8)	−0.3	0.757	5.3*	(2.7, 8.0)
	2006–2020	19.0*	(11.1, 27.4)	5.1	<0.001		

* Indicates that the APC and AAPC are significantly different from zero at the α = 0.05 level.

The comparison between Tables 4 and 6 highlights the diverse survival rates among gynecological cancers and their corresponding trends in mortality rates over time. Uterine corpus cancer demonstrates a relatively higher 5‐year survival rate compared to vulva cancer, indicating differences in prognosis. Cervical cancer exhibits a remarkable decline in annual mortality rates, reflecting substantial progress in its management. Similarly, uterine corpus and ovarian cancers show significant reductions in mortality rates over the study period, suggesting improvements in their treatment. Conversely, other female genital organ cancers exhibit a notable increase in mortality rates, emphasizing the necessity for further investigation and intervention.

## 4 DISCUSSION

Our study systematically analyses the gynecological cancer among elderly women of United States for the period 1975 to 2020 using joinpoint regression model. A study by Piechocki et al. revealed a correlation between age groups and the incidence and mortality rates of each assessed cancer, underscoring the importance of considering age‐specific factors in understanding and addressing cancer outcomes.^5^ A study by Cui et al.^14^ examines cancer incidence and survival rates in individuals aged 55 and older in the United States, crucial in aging societies like the United States. Utilizing data from the SEER database spanning 1975 to 2019, the study finds a decreasing trend in cancer incidence but increasing survival rates among this demographic. Notably, individuals aged 75 to 79 and 80 to 84 exhibit the highest cancer incidence rates.^17^ The observed fourfold surge in gynecological cancer diagnoses among elderly women from 1975 to 2020 is a notable trend, with uterine corpus cancers constituting the majority. However, despite the prevalence of uterine corpus cancers, the survival rates remain a concern, as only 23.5% of cases result in survival, and a substantial 76.5% face mortality, with 39.3% attributed to cancer. The temporal trend analysis elucidates a remarkable increase in key indicators, including incidence, overall mortality, cancer‐specific mortality, and mortality due to other causes over the study period.

The descriptive statistics underscore the evolving landscape of gynecological cancer incidence over the studied period, emphasizing the predominance of uterine corpus cancers and the critical impact on survival outcomes. The racial distribution further elucidates the diverse demographic representation within the study, shedding light on disparities that may influence cancer‐related outcomes. According to Somasegar et al.,^6^ uterine cancer mortality rates have risen since 2001 across all racial and ethnic groups, with the most substantial increase observed among younger non‐Hispanic Black and Hispanic women. A study by Cooley et al. ^15^ delves into the prevalence of late‐stage cervical cancer in women aged 65 and older, a demographic often overlooked in screening guidelines. Analyzing data from the California Cancer Registry spanning 2009 to 2018, it reveals that a substantial proportion of older women present with late‐stage disease compared to younger counterparts, leading to lower survival rates. Factors such as age, histologic subtypes, and comorbidities contribute to the increased risk of late‐stage diagnosis in this age group.^18^

The nuanced variations in trend of incidence underscore the dynamic nature of gynecological cancer incidence, emphasizing the importance of tailored and age‐specific approaches to intervention and screening efforts. Earlier, Adegoke et al. ^16^ reported the reductions in the overall and race‐specific incidence rates of invasive cervical cancer in the United States based on analysis of SEER data. Similar findings were reported by Moon et al. in Korea.^17^ They revealed that the incidence and mortality of cervical cancer have decreased since 1993 due to implementation of cancer screening programs and changes in lifestyle. In our results both vulva and vaginal incidence have increased during the period 1975 to 2020, which is contradicting to earlier findings by Zhou & Yue, where they reported that the increase in incidence of vulva cancer in the United States during 2001 to 2018 but decrease in the incidence of vaginal cancer.^18^ There is a declined trend in the incidence and mortality of ovarian cancer in the United States during the period 1975 to 2020, which corroborates with the previous findings by Malvezzi et al.^19^

Noteworthy variations in the incidence of gynecological cancers across age groups shed light on distinct patterns. Cervical cancer sees an increase in the 65 to 69 and 70 to 74 age brackets, while uterine corpus cancer rises significantly among women aged 85 and above. Ovarian cancer displays a decline in incidence for age groups under 79 but rises substantially in women aged 80 and above. Vaginal and vulvar cancers follow a similar pattern, with a decrease in incidence under 80 and a significant increase in those aged 80 and above. These age‐specific trends underscore the importance of tailoring preventive and management strategies to different age cohorts.

The analysis of gynecological cancer trends over different periods reveals significant insights into both incidence and mortality rates. The APC in incidence showed statistical significance during specific periods, emphasizing the dynamic nature of these cancers. Encouragingly, the overall mortality rate displayed a significant decline over the study duration, accompanied by notable reductions in mortality rates due to cancer and other causes. These positive trends suggest advancements in detection, treatment, and overall health care.

According to A. O. Knudsen et al., gynecological cancer exhibits age‐dependent mortality rates and survival, indicating a significantly shorter survival period among the elderly population.^4^ The 5‐year survival rates presented in the table further highlight the heterogeneity in outcomes among various gynecological cancers in elderly women. It underscores the importance of understanding and addressing the unique challenges posed by different gynecological cancers. The trends in cancer‐specific mortality rates provide additional insights, with cervical, uterine corpus, and ovarian cancers displaying significant declines over the study period. However, the rise in mortality rates for other female genital organs during 2006 to 2020 warrants attention and further investigation. These findings collectively contribute to the body of knowledge surrounding gynecological cancers among elderly women, guiding future research directions and informing targeted interventions to improve outcomes in this population.

The strength of our analysis lies in the most recent available SEER population‐based registry data and it also provides a detailed overview of gynecological cancer epidemiology, particularly in elderly women. It is worth noting here the limitation of our study that the registry data did not reveal the causes for change in documentation.

## 5 CONCLUSION

This comprehensive analysis of gynecological cancer trends among a substantial cohort of elderly women spanning 1975 to 2020 illuminates critical insights into incidence, survival rates, and mortality patterns. The observed fourfold surge in diagnoses underscores the evolving landscape of these cancers, with uterine corpus cancers notably prevalent. Despite advancements in health care, survival rates vary significantly among different gynecological cancers, emphasizing the urgency for tailored preventive and management strategies. Encouragingly, the declining overall mortality rate and cancer‐specific mortality rates for cervical, uterine corpus, and ovarian cancers signify positive strides in detection and treatment. However, the persistent challenges and increasing mortality rates for cancers in other female genital organs during the most recent period highlight the need for continued research and targeted interventions. These findings collectively contribute valuable insights to the field, guiding future efforts to enhance the understanding and management of gynecological cancers in the elderly population.

## AUTHOR CONTRIBUTIONS

Conception & design of study: SP, PKS. Acquisition of data: SP. Data analysis and/or interpretation: SP, DJ, SoP. Drafting: SP, PKS, KA, DJ. Critical revision of manuscript: PKS. Approval of final version of manuscript: all authors.

## FUNDING INFORMATION

None.

## CONFLICT OF INTEREST STATEMENT

The authors have no conflicts of interest to declare.
